# Development and validation of a predictive model for weight loss psychological distress in obese patients: a cross-sectional study

**DOI:** 10.3389/fpubh.2025.1700100

**Published:** 2025-12-01

**Authors:** Zhiqiang Cheng, Meiling Liu, Xiaxin Li, Jiazhen Tang, Caihong Huang

**Affiliations:** 1 Department of Health Management Medicine, The First Affiliated Hospital of Nanchang University, Nanchang, Jiangxi, China; 2 Department of Nursing, The Affiliated Hospital of Jiangxi University of Chinese Medicine, Nanchang, Jiangxi, China; 3 Department of Anesthesiology and Surgery, The First Affiliated Hospital of Gan Nan Medical University, Jiangxi, Ganzhou, China

**Keywords:** obesity, psychological distress, predictive model, nursing care, risk factors

## Abstract

**Background:**

Obese patients are more likely to experience psychological distress symptoms, such as low self-esteem, anxiety and depression. Approximately 18–55% of obese patients will encounter problems such as abdominal distension, purple skin lines and facial acne due to changes in their body shape and appearance. Establishing a predictive model for psychological distress in obese patients during the weight loss process, early identification of high-risk groups, and the adoption of proactive intervention measures can help reduce the incidence of psychological distress.

**Aims:**

Explore the risk factors for psychological distress among middle-aged and young obese patients, and construct and validate a risk prediction model for psychological distress.

**Methods:**

The study was a cross-sectional survey study, From January to June in 2025, a total of 357 obese patients were selected from 22 tertiary hospitals in Jiangxi Province, China. The model was constructed by univariate and logistic regression analyses. They were divided into the group with significant psychological distress (K10 ≥ 16 points) and the group without significant psychological distress (K10 < 16 points). R4.2.3 Statistical software was used to construct a risk prediction model for psychological distress in obese patients regarding weight loss. The discriminative ability of the model was evaluated by the receiver operating characteristic (ROC) curve, the accuracy of the predictive model was evaluated by the Hosmer–Lemeshow test and calibration curves, and the clinical utility was assessed using decision curve analysis (DCA).

**Results:**

Two hundred fifty-five patients were in the training set and 102 were in the validation set. Through logistics regression analysis, the following seven predictive factors were obtained: age (youth), gender (female), history of chronic diseases, high BMI, weight loss method (diet and exercise for weight loss), perceived social support, and general self-efficacy. The area under the ROC curve of the model was 0.852 (95%*CI*, 0.806 ~ 0.897). The sensitivity and specificity were 0.804 and 0.729. The maximum Youden index was 0.520, and the best cut-off value was 0.396. The Hosmer–Lemeshow test showed that *χ*^2^ = 4.560 and *p* = 0.803. The internal and external validation results showed that the area under the ROC curve was 0.858 and 0.833 respectively, and the Hosmer–Lemeshow test results showed that the *χ*^2^ was 0.664 and 0.765, respectively. The decision curve analysis shows that obese patients have better clinical benefits.

**Conclusion:**

This study developed a predictive model for weight loss psychological distress in obese patients, which has strong predictive performance and has been verified by internal and external cohorts. It was helpful for the early detection of high-risk groups for weight loss psychological distress.

## Background

Obesity was a chronic and recurrent disease caused by multiple factors such as genetics, social economy and lifestyle, characterized by excessive accumulation of fat in the body, accompanied by organ dysfunction and decreased ability to perform daily activities (1). The main parameter for diagnosing obesity was the body mass index BMI (in kg/m^2^). The ranges of 18.5 ~ 24.9 for normality was considered overweight, 25 ~ 29.9 for overweight, and ≥30 for obesity (2). Up to 2022, there were a total of 2.5 billion overweight people worldwide, among which 890 million were obese. In 147 countries, the proportion of obese people in the total population exceeded 50% (3). It was expected that by 2030, the global obese population will reach about 1.1 billion, of which 487 million were men and 643 million were women, and the number of overweight and obese people in China will reach 515 million (4). Obesity has become one of the important risk factors for cardiovascular and cerebrovascular diseases, diabetes, chronic kidney disease and other diseases. According to a cancer survey in China, 48.47% of cancer patients among 14.14 million people were related to obesity, which has become a risk factor for many types of cancer (5). Obesity was a driving factor for the aggravation of chronic kidney disease, which can easily lead to glomerular hypertrophy, accompanied by focal and segmental glomerular sclerosis, and eventually develop into uremia (6).

Previous studies have shown that obese patients, due to their bloated bodies, have purple wrinkles on their skin and acne on their faces. Approximately 18 to 55% of obese patients were prone to psychological distress symptoms such as low self-esteem, anxiety, depression and fear (7, 8). Psychological distress was an unpleasant psychological experience caused by multiple factors, mainly manifested in psychological, social and physical states (9). Psychological distress was prone to cause eating disorders in obese patients, such as bulimia nervosa and bulimia nervosa (10). Research has found that there was also a certain relationship between obesity and anxiety and depression. A higher weight increases the risk of depression in obese patients (11). Weight shame was also one of the manifestations of psychological distress. From a social point of view, obese patients were often regarded as representatives of laziness and lack of self-discipline, and they were susceptible to ridicule and bullying in employment, education, and health care (12).

The psychological distress of obese patients was influenced by multiple factors. Previous studies have shown that gender was an important factor influencing the psychological distress of obese patients. A cross-sectional survey in Sweden involving 413 obese patients revealed that over one-third of female obese patients had symptoms of anxiety or depression, the risk of anxiety and depression among female obese patients was 5.65 times that of male patients (13). Compared with men, female patients pay more attention to their appearance and figure. Obesity can seriously affect the marriage and daily social activities of unmarried women (14).

Studies have shown that there was a significant relationship between a higher BMI and psychological distress. The epidemic rate of obesity varies in different regions. The overall prevalence of global obesity was 41.5%. It is more prevalent among older patients, female patients, and urban residents. Regarding regional differences, the prevalence rate in Latin America is around 55.1%, and in Central America it was 52.9%. The rate of obesity among young patients aged 15–40 has increased the fastest, rising from 16.3 to 33.6% (15). BMI increases with age, peaks between the ages of 50 and 59, and gradually declines after the age of 60. The higher the body mass index, the more difficult it is to lose weight, and the more time and medical expenses it takes.

The social support level was a protective factor against psychological distress. Social support comes from various aspects such as family, friends, and colleagues, and can help patients reduce the impact of negative emotions and enhance their resilience in dealing with hardships (16). A large number of studies have shown that a lack of social support can lead to estrangement between patients and their families and friends, increasing the risk of depression and suicide (17). Previous studies have shown that self-efficacy was another protective factor for psychological distress. Self-efficacy was a patient’s belief in their ability to complete a certain task or behavior (18). The higher the level of self-efficacy of a patient, the greater their confidence in completing a certain task and the less they are affected by negative emotions. For patients trying to lose weight, the key lies in how to provide multi-faceted support to obese patients, boost their confidence and help them achieve their weight loss goals (19).

The above research indicates that the psychological distress experienced by obese patients during the weight loss process was influenced by various factors, such as age, gender, BMI, and social support. However, it is still unclear to what extent these factors can predict the probability of psychological distress occurring in obese patients during the weight loss process. This study aims to build a prediction model by incorporating relevant risk factors and protective factors, and to visualize the model through a nomogram graph. This will enable regular assessment to identify the psychological distress of obese patients at an early stage, and help healthcare professionals formulate targeted intervention measures. Therefore, this study proposes three hypotheses:

*Hypothesis 1*: Gender has a predictive effect on the psychological distress of obese patients.

*Hypothesis 2*: BMI has a predictive effect on the psychological distress of obese patients.

*Hypothesis 3*: Social support and self-efficacy have a protective effect on the psychological distress of obese patients.

## Methods

### Study design

The study employed a cross-sectional survey design.

### Settings and participants

From January to June 2025, a convenience sampling method was used to randomly select obese patients from a tertiary hospital in Jiangxi Province, China to participate in an anonymous survey. The samples selected from January to May 2025 constituted the training set, while the obese patients from another tertiary hospital in Jiangxi Province selected in June 2025 formed the validation set. The patients were divided into the significantly distressed group (K10 ≥ 16 points) and the non-significantly distressed group (K10 < 16 points) based on the level of psychological distress. Inclusion criteria: ① Age: 18 ~ 60 years old, BMI: ≥ 30 kg/m^2^; ② First-time hospital visitors and communicate normally; ③ Signed the informed consent form and agreed to participate in this study. Exclusion criteria: ① Have a history of mental or psychiatric disorders; ② Patients with severe cardiovascular or cerebrovascular diseases or malignant tumors; ③ Withdraw from this study halfway. This study identified a total of 16 risk factors through preliminary investigations and literature reviews. The sample size required for the EPV (events per variable) calculation was determined. In this model construction, it is expected to include 7–10 predictor variables (20). According to the research results of Steptoe (8), the incidence of psychological distress due to weight loss among obese patients was approximately 40%. Considering a 20% sample attrition rate, the required number of samples for the training set is 7 × 10/0.4/0.8 = 219.the sample size required for the validation set was 1/4 to 1/2 of that of the training set (55 ~ 109).

### Questionnaire collection procedure

The research team consisted of 3 personnel from the weight management center and 2 graduate students in nursing. Before the start of the investigation, the research group received unified training and obtained the consent of the department head. The investigation was conducted after the distribution of questionnaires. Before distributing the questionnaires, the researchers explained the purpose, content and precautions of the investigation to the participants. The questionnaire survey was conducted using Questionnaire Star to distribute electronic questionnaires. The following sub-questionnaires have all been translated into Chinese and have good reliability and validity. The participants were guided to fill out the questionnaire using a unified instruction, and the filling was done anonymously to protect the privacy of the participants. The time taken to complete the questionnaire was 10 ~ 12 min. After the completion of the questionnaire, it was checked by two members of the research team to eliminate incomplete or incorrect questionnaires, ensuring the accuracy of the data. A total of 365 questionnaires were filled out, and 357 valid questionnaires were recovered, with an effective recovery rate of 97.81%.

### Ethical considerations

This study has been approved by the Neighborhood Committee of The First Affiliated Hospital of Nanchang University (IIT2025-055). All participants were informed of the content and purpose of the study and signed the informed consent form. The study does not involve the participants’ names, ID numbers, home addresses, or any other personal information. The privacy of the participants was strictly protected. The data will not be used for any commercial purposes and will only be reported in summary form.

### Measures

#### Data collection tool

##### General social survey questionnaire

The questionnaire was designed by the research team itself. Its contents mainly include age, gender, educational level, occupation, monthly income, history of chronic diseases, marital status, and medical insurance payment situation.

##### Perceived social support scale (PSSS)

This scale was developed by Zimet and was mainly used to assess the extent to which patients receive support from family members, friends and other sources (21). The scale consists of three dimensions: family support, friend support and other support, with 12 items. Each item ranges from “strongly disagree” to “strongly agree,” and was scored from 1 to 7 points. The total score ranges from 12 to 84. The overall Cronbach’s alpha coefficient of this scale was 0.934, and the Cronbach’s alpha coefficients of each dimension were 0.845, 0.902 and 0.876, indicating good reliability and validity. The higher the score, the higher the level of social support received by the patient. In this study, the Cronbach’s alpha coefficient of this scale was 0.884.

##### The brief illness perception questionnaire (BIPQ)

This scale was consists of 3 dimensions and 9 items, the Cronbach’s *α* coefficient was 0.893, indicating good reliability and validity (22). The cognitive dimension has 6 items: influence, disease course, individual control, treatment control, symptoms, and identity, which were used to measure patients’ cognition of the disease; the emotional dimension has 2 items: disease concern and emotion, which were used to measure patients’ emotional experiences. The understanding dimension has 2 items, one measuring the degree of patients’ understanding of the disease and the other an open-ended question item. Except for the open-ended item, the scores of other items range from 0 to 10 (where the scores of items 3, 4, and 7 were calculated in reverse), and the total score ranges from 0 to 80. The higher the score, the deeper the negative feelings of the patients toward the disease. In this study, the Cronbach’s *α* coefficient of this scale was 0.865.

##### Kessler psychological distress scale (K10)

This scale was consists of 10 items and was mainly used to assess the frequency of symptoms related to specific mental health conditions such as anxiety and stress levels experienced by patients in the past 4 weeks (23). Each item of the K10 scale was scored on a 5 point scale, with each item ranging from 1 to 5 points. The higher the total score, the worse the patient’s mental health condition. With scores of 10 ~ 15 indicating Good mental health status; with scores of 16 ~ 21 indicating average mental health status; with scores of 22 ~ 29 indicating poor mental health status; with scores of 30 ~ 50 indicating very poor mental health status. The split-half reliability of the K10 scale was 0.7076, and the Cronbach’s *α* coefficient was 0.801, indicating good reliability and validity. In this study, the Cronbach’s α coefficient was 0.826.

##### General self-efficacy scale (GSES)

The Cronbach’s α coefficient of this scale was 0.872, and the test–retest reliability was 0.836. The scale consists of 10 items, each using the Likert 4-point rating method, ranging from “completely incorrect” to “completely correct,” with scores ranging from 1 to 4. The total score ranges from 10 to 40, with higher scores indicating stronger self-confidence (24).

### Data analysis

The data were statistically analyzed using SPSS 25.0. Measurement data that followed a normal distribution were described by mean and standard deviation, and comparisons between groups were conducted using *t*-tests and analysis of variance; measurement data that did not follow a normal distribution were expressed as M (P25, P75), and statistical analysis was performed using the Mann–Whitney *U* test; count data were represented by frequency and percentage, and statistical analysis was conducted using the *χ*^2^ test or Fisher’s exact probability method. The independent variables with statistical significance identified in the single-factor analysis were subjected to multicollinearity diagnosis. When the variance inflation factor (VIF) was <5, it indicated that there was no multicollinearity among the independent variables. The independent variables with statistical significance were included in the binary Logistic regression analysis. R4.2.3 was used to constructed a nomogram prediction model. The training set data was internally validated using 1,000 Bootstrap repeated sampling, and the external validation was performed using the validation set data. The area under the ROC curve was used to evaluate the discrimination of the model, the calibration curve and Hosmer–Lemeshow test was used to evaluate the calibration of the model, the DCA was used to evaluate the clinical effectiveness, and *p* < 0.05 was considered statistically significant.

## Results

### General information of middle-aged and young obese patients

A total of 260 patients were included. Among them, 5 patients withdrew from the study due to personal reasons. Finally, 255 patients were included, with 120 males and 135 females, aged 18 to 59 years. The sample collection was conducted in two hospitals in Jiangxi Province, China. However, the obese patients came from five different provinces. Jiangxi Province (144), Hunan Province (45), Anhui Province (28) Shandong Province (22), and Hubei Province (16). Based on a psychological distress score of 4 as the critical value, 107 patients (41.96%) had significant psychological distress (≥4 points). There were significant statistical differences between the two groups in terms of age, gender, history of chronic diseases, monthly income level, BMI, waistline, weight loss methods, PSSS and GSES (*p* < 0.05) (Table 1).

**Table 1:** Sociodemographic characteristics of the participants and Single factor analysis of psychological distress in obese patients (*n* = 255, Jiangxi, China).

Variables	K10 < 16 (*n* = 148)	K10 ≥ 16 (*n* = 107)	Test statistics	*P*
Age (in years)	35.00 (33.00, 42.00)	33.00 (25.00, 39.00)	3.712^b^	<0.001
Gender			6.928^a^	0.008
Male	80 (54.05%)	40 (37.38%)		
Female	68 (45.95%)	67 (62.62%)		
Educational background			0.936^a^	0.817
High school and below	15 (10.14%)	10 (9.35%)		
College degree	43 (29.05%)	26 (24.30%)		
Bachelor’s degree	75 (50.67%)	58 (54.20%)		
Master’s degree	15 (10.14%)	13 (12.15%)		
Employment status			0.024^a^	0.887
Unemployed	72 (48.65%)	51 (47.66%)		
Employed	76 (51.35%)	56 (52.34%)		
Marital status			0.331^a^	0.565
Single	69 (46.62%)	46 (42.99%)		
Married	79 (53.38%)	61 (57.01%)		
Residence			3.259^a^	0.071
Rural	46 (31.08%)	45 (42.06%)		
Urban	102 (68.92%)	62 (57.94%)		
Chronic disease			9.989^a^	0.002
No	85 (57.43%)	40 (37.38%)		
Yes	63 (42.57%)	67 (62.62%)		
Monthly income			12.886^a^	0.005
<4000yuan	35 (23.65%)	44 (41.12%)		
4,000 ~ 6000yuan	59 (39.86%)	40 (37.38%)		
>6000yuan	54 (36.49%)	23 (21.50%)		
Primary insurance status				
No	19 (12.84%)	13 (12.15%)	0.203^a^	0.977
NRCMS	48 (32.43%)	33 (30.84%)		
URBMI	43 (29.05%)	31 (28.97%)		
UEBMI	38 (25.68%)	30 (28.04%)		
BMI (Kg/m^2^)	30.11 (28.42, 33.24)	32.62 (28.89, 36.55)	−3.268^b^	0.001
Waistline (cm)	101.00 (98.00, 105.75)	102.00 (100.00, 110.00)	−2.116^b^	0.034
Weight loss goal			0.020^a^	0.990
<5%	60 (40.54%)	28 (26.17%)		
5% ~ 10%	30 (20.27%)	22 (20.56%)		
11% ~ 15%	58 (39.19%)	57 (53.27%)		
Weight loss methods			17.289^a^	<0.001
Metabolic/bariatric surgery	60 (40.54%)	24 (22.43%)		
Anti-obesity drugs	59 (39.87%)	38 (35.51%)		
Diet and exercise for weight loss	29 (19.59%)	45 (42.06%)		
PSSS	56.00 (48.00, 66.00)	43.00 (36.00, 52.00)	−7.261^b^	<0.001
BIPQ	39.50 (35.00, 43.00)	42.00 (35.00, 46.00)	−2.141^b^	<0.032
GSES	23.50 (20.00, 28.00)	22.00 (19.00, 26.00)	−2.099^b^	<0.036

a = χ^2^; b = Z; K10, Kessler psychological distress scale; NRCMS, New Rural Cooperative Medical Scheme; URBMI, Urban Residents Basic Medical Insurance; UEBMI, Urban Employees Basic Medical Insurance; PSSS, Perceived Social Support Scale; BIPQ, The Brief Illness Perception Questionnaire; GSES, General Self-Efficacy Scale.

### Logistic regression analysis of psychological distress related to weight loss in middle-aged and young obese patients

Taking the 10 factors with statistical significance from the univariate analysis as independent variables, and setting the presence of psychological distress (K10 ≥ 16 points) in obese patients as the dependent variable for logistic regression analysis, the assignment of independent variable values were shown in Table 2. The VIF of the above 10 risk factors was all less than 5, and there was no multicollinearity. The results of the logistic regression analysis showed that for obese patients, being young, female, having chronic diseases, an increase of one unit in BMI, a decrease of 1 point in PSSS, and a decrease of 1 point in GSES were independent risk factors for psychological distress related to weight loss. The specific situation were shown in Table 3.

**Table 2:** Table of variable assignments (*n* = 255, Jiangxi, China).

Variables	Method of assignment
Ages	Original value
Gender	Male = 1, Female = 2
Chronic disease	No = 1, Yes = 2
Monthly income	<4,000 = 1, 4,000 ~ 6,000 = 2, >6,000 = 3
Weight loss goal	<5% = 1, 5% ~ 10% = 2, 11% ~ 15% = 3
Weight loss methods	Metabolic/bariatric surgery = 1, Anti-obesity drugs = 2, Diet and exercise for weight loss = 3
BMI	Original value
Waistline	Original value
PSSS	Original value
BIPQ	Original value
GSES	Original value

**Table 3:** Logistic regression analysis results of the psychological distress caused by weight loss in obese patients (*n* = 255, Jiangxi, China).

Variables	*β*	SE	Wald	*P*	OR	95% *CI*	
						LLCL ULCL	
Constant	1.862	1.793	1.078	0.299	6.437		
Gender	0.955	0.326	8.599	0.003	2.598	1.372	4.917
Ages	−0.967	0.341	8.034	0.005	0.38	0.195	0.742
Chronic disease	1.142	0.324	12.386	<0.001	3.133	1.659	5.918
Weight loss methods	1.001	0.346	8.396	0.004	2.722	1.383	5.358
BMI	0.109	0.047	5.469	0.019	1.115	1.018	1.222
PSSS	−0.088	0.015	35.086	<0.001	0.916	0.889	0.943
GSES	−0.096	0.03	10.103	0.001	0.908	0.856	0.964

### Constructing a nomogram for psychological distress in obese patients undergoing weight loss

**Figure 1:**
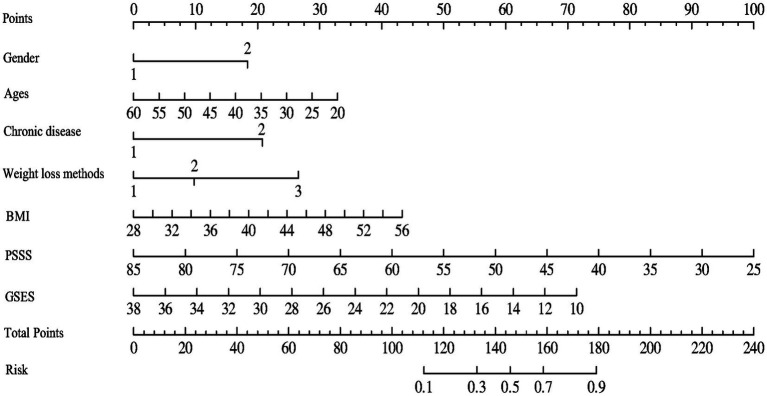
Nomogram for predicting the psychological distress of obese patients during weight loss.

The nomogram for predicting psychological distress in obese patients during weight loss was constructed based on the results of Logistic regression analysis, as shown in Figure 1. Calculation method for the risk of psychological distress in obese patients during weight loss: Firstly, find the corresponding score for each predictor factor, then add up all the scores to obtain the total score, and finally calculate the probability of psychological distress in obese patients based on the score on the risk axis.

### The predictive performance of the prediction model for psychological distress in obese patients during weight loss

**Figure 2:**
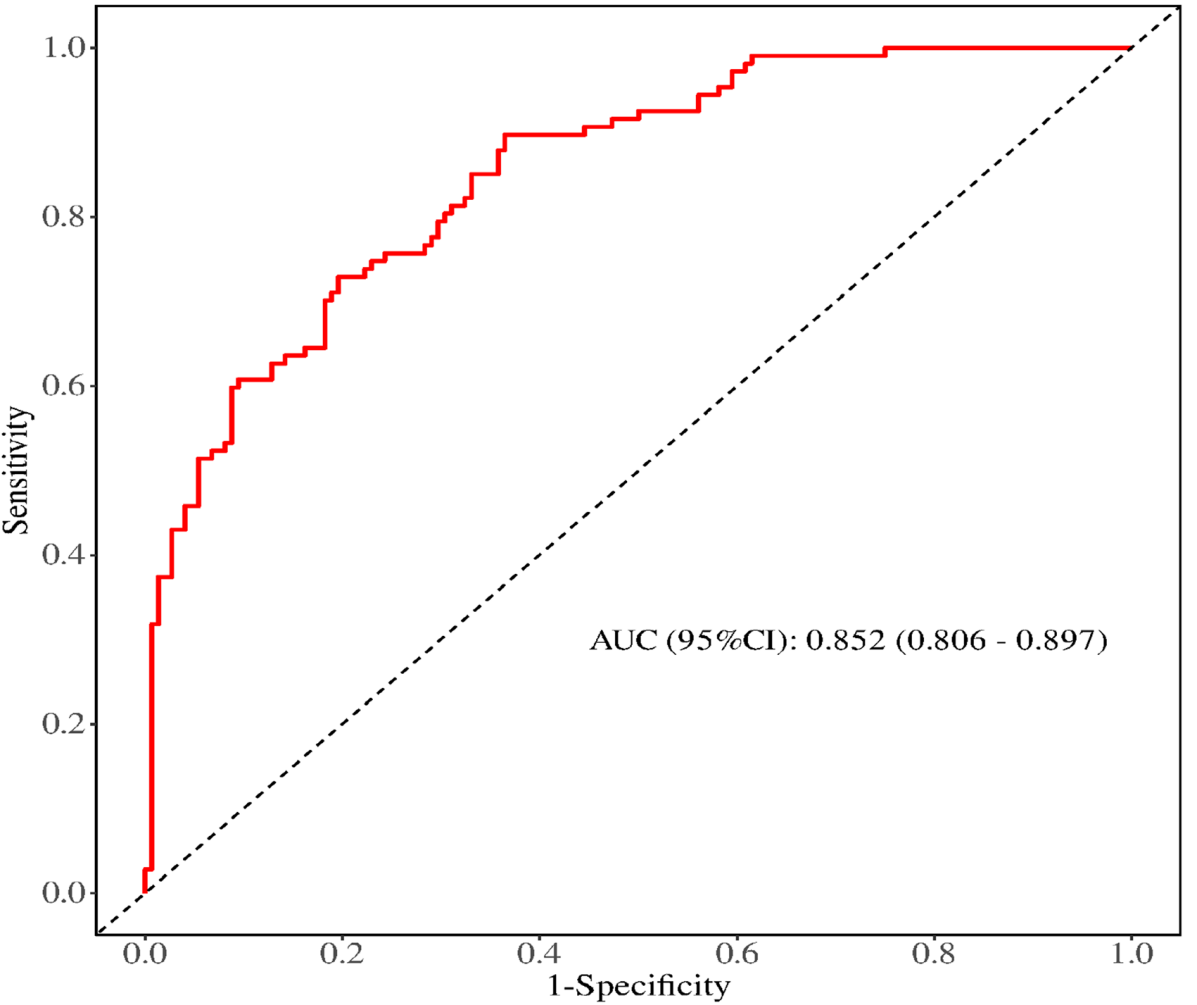
The ROC curve for predicting the psychological distress of weight loss in obese patients.

**Figure 3:**
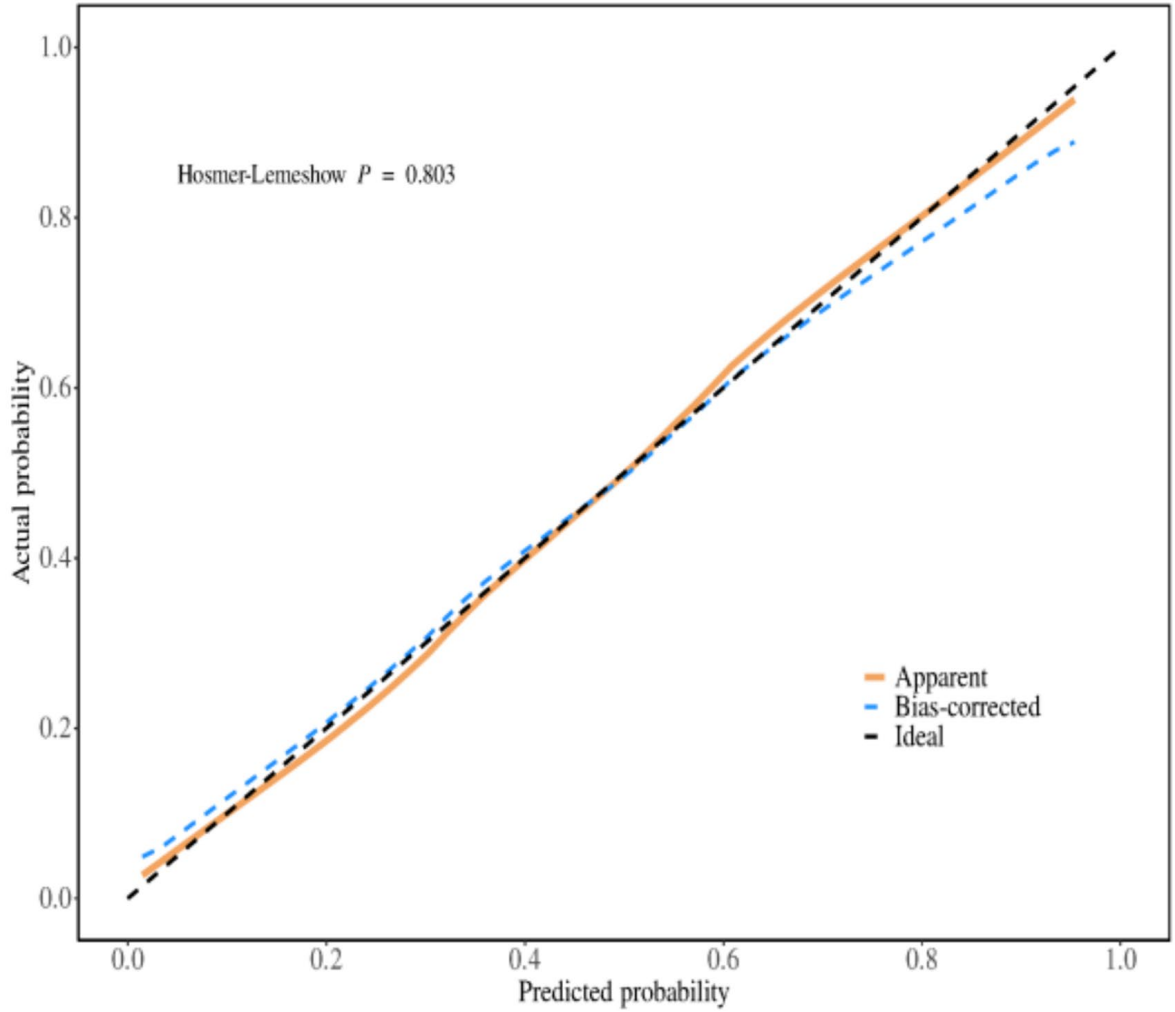
The calibration curve of the prediction model for the psychological distress of weight loss In obese patients.

The receiver operating characteristic (ROC) curves was used to evaluate the discrimination ability of the prediction model, the area under the ROC curve was 0.852 (95% *CI*, 0.806 ~ 0.897), as shown in Figure 2. The maximum Youden index was 0.520, the critical value was 0.396, the sensitivity was 0.804, and the specificity was 0.729. The calibration curve was used to assess the calibration ability of the prediction model, as shown in Figure 3. The Hosmer–Lemeshow test showed that *χ*^2^ = 4.560, *p* = 0.803 (*p* > 0.05).

### The internal and external validation of the prediction model for the psychological distress of weight loss in obese patients

#### Internal validation

**Figure 4:**
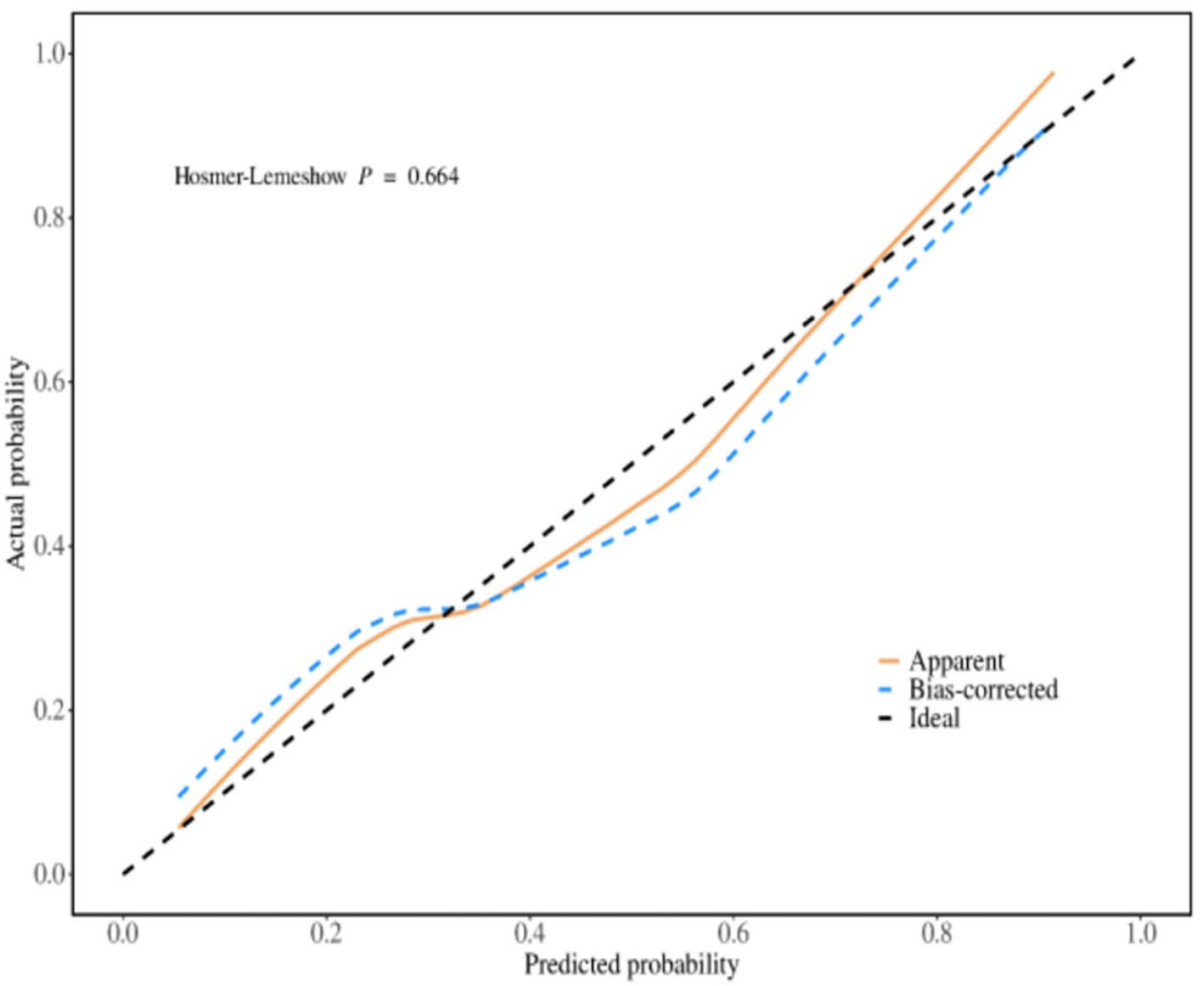
Internal verification calibration curve.

The predictive performance of the evaluation model was evaluated by repeating sampling using the Bootstrap method 1,000 times. The results showed that the area under the ROC curve was 0.858 (95% *CI*, 0.806 ~ 0.910), the sensitivity was 0.872, and the specificity was 0.778. The Hosmer–Lemeshow test indicated that *χ*^2^ = 5.849, *p* = 0.664 (*p* > 0.05), and the calibration curve graph showed that the degree of fit between the calibration curve and the ideal curve was high. The calibration curve was shown in Figure 4.

#### External validation

**Figure 5:**
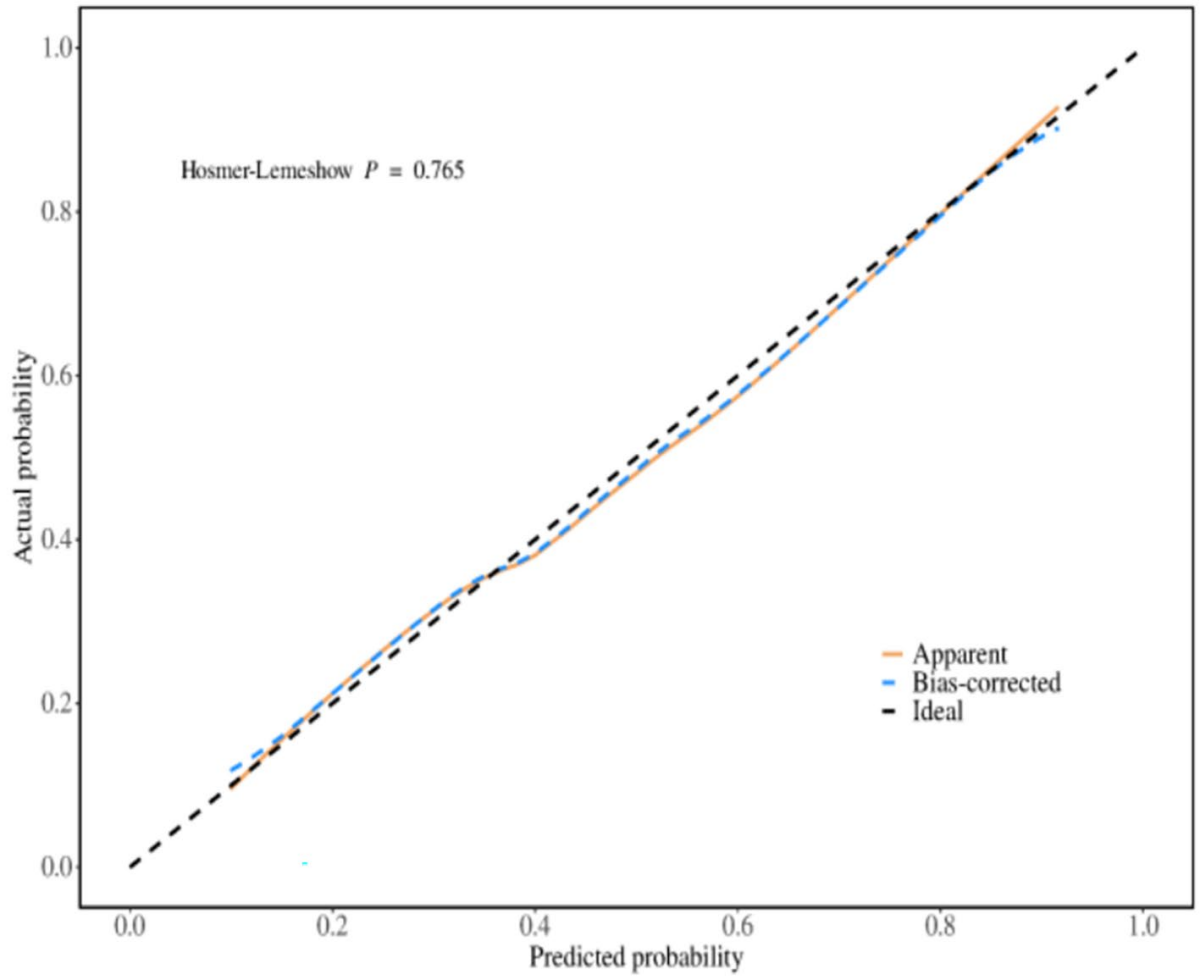
External validation calibration curve.

**Figure 6:**
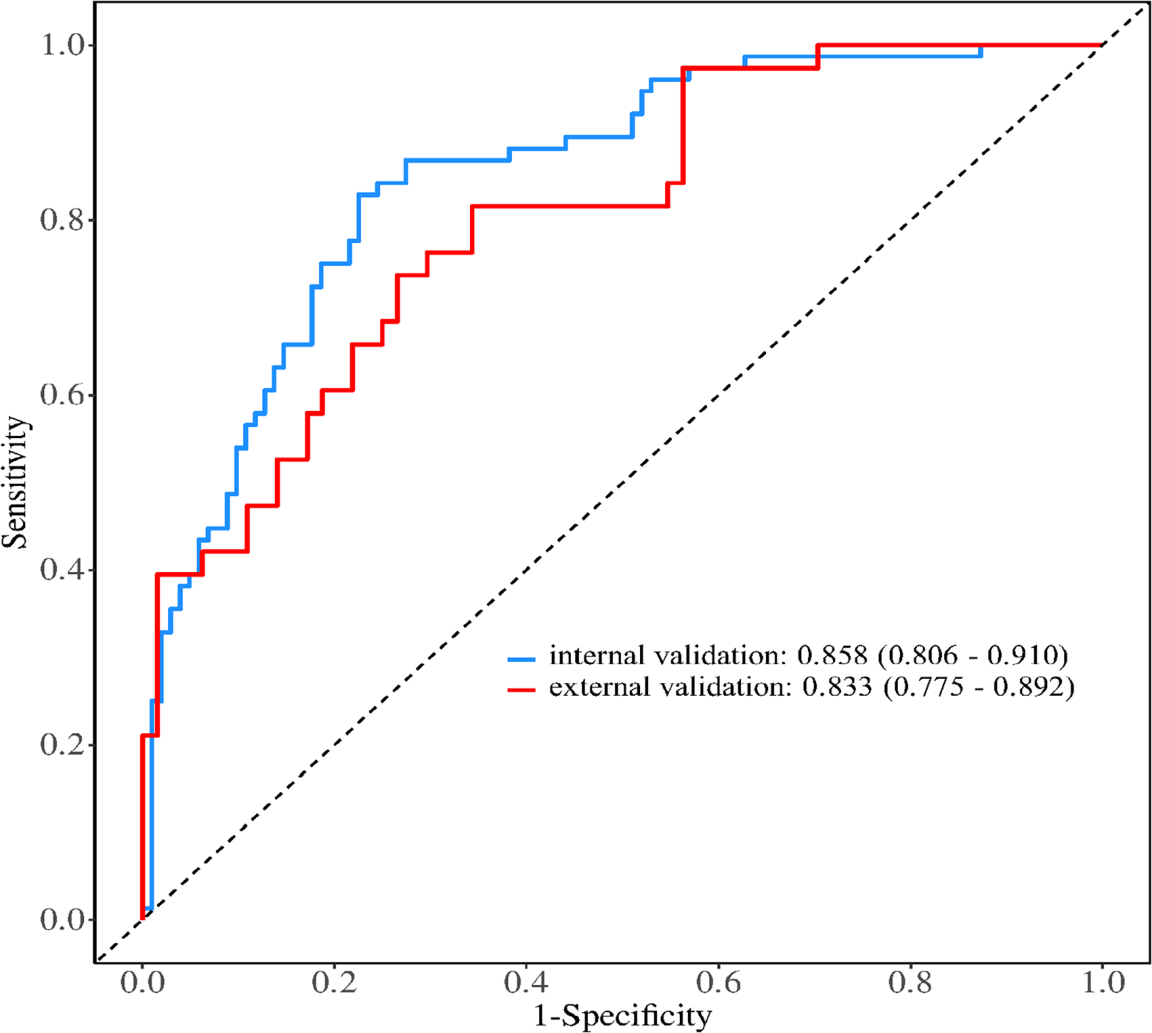
ROC curves for internal validation and external validation.

A total of 102 patients were included in the external validation. Among them, 48 were male and 54 were female, 39 cases (38.24%) had significant psychological distress (K10 ≥ 4), aged 18–58 years (average age = 33.52 ± 10.01). The general data of the modeling set and the validation set showed that there were no statistically significant differences between the two groups in terms of age, gender, history of chronic diseases, weight loss methods, BMI, PSSS, and GSES (*p* > 0.05). The external validation results showed that the area under the ROC curve was 0.833 (95% *CI*: 0.775 ~ 0.892), the sensitivity was 0.851, and the specificity was 0.772. The Hosmer–Lemeshow test showed that *χ*^2^ = 4.926, *p* = 0.765 (*p* > 0.05). The external validation calibration curve was shown in Figure 5. The ROC curve for internal validation and external validation was shown in Figure 6.

### The decision curve analysis

**Figure 7:**
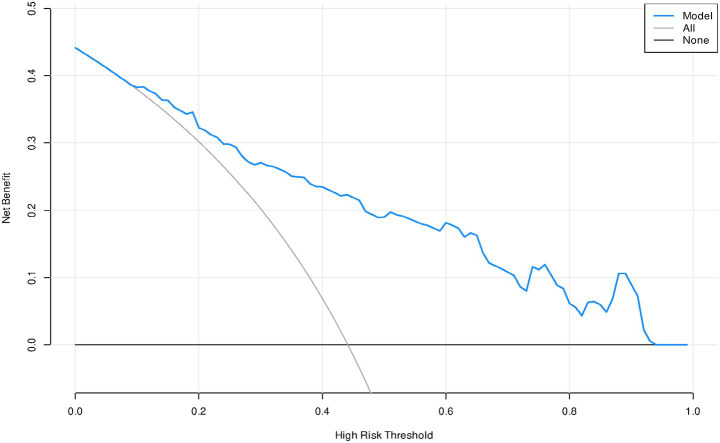
The decision curve analysis of the prediction model for the psychological distress of weight loss in obese patients.

The decision curve analysis was drawn. The “None” line indicates that no psychological distress occurred in all obese patients, the “All” line indicates that psychological distress occurred in all obese patients, and the “Model” line represents the probability predicted by the model for psychological distress in obese patients. The results of the decision curve analysis showed that the prediction curve was close to the upper right corner, suggesting that the model has good clinical effectiveness (Figure 7).

### Analysis of predictive factors for the psychological distress of weight loss in obese patients

The psychological distress caused by weight loss was more severe among young and female obese patients. The psychological stress caused by weight loss was more severe among young and female obese patients. Ngenge’s study also showed that female obese patients have a higher risk of experiencing psychological problems such as anxiety and depression compared to male patients (13). Compared with older and male patients, young and female patients pay more attention to their weight loss results during the weight loss process. When the weight loss goal cannot be achieved and no significant improvement in body shape and appearance was observed, young women were more likely to experience negative emotions such as anxiety, worry, and depression, thereby exacerbating the psychological distress during the weight loss process (25). In this study, for every 1 kg/m^2^ increase in MBI, the risk of experiencing psychological distress due to weight loss increases by 1.115 times. Macho et al.’s (26) research also shows that as BMI increases, obese patients suffer more discrimination, have a higher level of stigma, psychological distress and depression. Previous studies (27) have shown that patients with a higher body mass index have lower satisfaction with their appearance. Patients with a larger body weight face greater difficulty in losing weight and need to persist for a long time, which makes them prone to psychological problems such as anxiety and depression. The results of this study revealed that compared to the Metabolic/bariatric surgery, patients who adopted diet and exercise for weight loss had a higher level of psychological distress. Studies have shown that the weight loss effect through Metabolic/bariatric surgery was more significant, and it can effectively reduce body weight in a short period of time. However, The Diet and exercise for weight loss method requires patients to persist for a long time and the effect was not obvious. Patients were prone to lose confidence in continuing weight loss, thus developing negative emotions and exacerbating their psychological distress (28, 29).

Obese patients with chronic diseases such as hypertension and diabetes have a 3.133 times higher risk of experiencing psychological distress during weight loss compared to obese patients without chronic diseases, which was similar to the results of Gallo et al. (30). Obese patients with cardiovascular and cerebrovascular diseases, diabetes, and chronic kidney disease were prone to increase the burden on their heart, kidneys and other body organs during weight loss. These patients were likely to suffer from complications such as heart failure and excessive fluctuations in blood sugar, which can hinder their weight loss progress and exacerbate psychological distress (31). Powell-Wiley et al.’s (32) study also confirmed that obese patients with concurrent chronic diseases have an increased incidence of cardiovascular events during the weight loss process, and this has a significant impact on the quality of life and prognosis of obese patients in the later stage. For obese patients with concurrent chronic diseases, various weight loss methods such as medication, surgery, diet, and exercise can be combined. Under the condition of controlling patients’ blood sugar, blood pressure, and blood lipids, following the principle of gradual progress, these methods can help patients alleviate negative emotions such as tension and anxiety, thereby achieving the weight loss goal.

The higher the level of PSSS, the lower the possibility that obese patients will experience psychological distress. When obese patients receive support from family members, friends and colleagues during the weight loss process, especially emotional encouragement and financial assistance, it can effectively alleviate the psychological pressure of the patients during the weight loss process. Patients lose weight together with multiple companions, it is more likely to stimulate the potential and confidence of the patients to lose weight, helping them complete the weight loss process better (33). When obese patients receive emotional and financial support from family members, friends and colleagues during their weight loss process, they can effectively cope with the problems encountered during the weight loss journey, enhance their confidence in losing weight, and effectively reduce the psychological pressure of weight loss (34).

Patients with lower levels of GSES are more likely to experience psychological distress. Self-efficacy refers to an individual’s confidence in completing a certain task or achieving a goal. The higher the level of self-efficacy, the greater the likelihood that the patient will be able to accomplish that task (35). Palmeira et al. (36) explored the relationship between GSES and psychological distress. By observing the weight loss outcomes of 1,627 obese patients over a period of 12 months, the results showed that obese patients with higher GSES scores had more significant weight loss, higher satisfaction with their body shape, and lower anxiety and depression scores. Moreover, these obese patients with higher GSES scores had a stronger willingness to persist in weight loss.

### The predictive model for psychological distress in obese patients has excellent predictive performance

We constructed a model based on relevant predictive factors and drew a type chart to predict the psychological distress experienced by obese patients during weight loss. The area under the ROC curve was used to evaluate the discrimination ability of the prediction model. When the area under the ROC curve is between 0.5 and 0.7, it indicates that the model’s discrimination ability is poor. The area under the ROC curve is between 0.7 and 0.9, the discrimination ability is good. The area under the ROC curve is greater than 0.9, it indicates that the model’s discrimination ability is high. In this study, the area under the ROC curve of the constructed prediction model is 0.852, the maximum Youden index is 0.520, the critical value is 0.396, the sensitivity is 0.804, and the specificity is 0.729. The results of internal validation and external validation show that the areas under the ROC curves of the prediction model are 0.858 and 0.833, respectively. The above results all indicate the discrimination ability of the prediction model is good. The calibration curve is used to evaluate the calibration ability of the prediction model. The calibration curve of this study shows a high degree of agreement between the predicted probability and the actual probability. The Hosmer–Lemeshow test indicates that the *P* > 0.05, indicating no statistical difference and suggesting that the calibration ability of the prediction model is good. The decision curve analysis indicates that the predictive model constructed in this study is clinically useful.

## Conclusion

This study investigated the factors that might affect the psychological distress of obese patients in Jiangxi Province, China, and constructed a nomogram for the predictive model. This model has excellent discrimination ability, calibration ability and clinical utility. It can assist clinical nurses in assessing the risk of psychological distress in obese patients during the weight loss process, thereby providing a reference basis for conducting prospective interventions.

### Relevance for clinical practice

Being young, female, having a history of chronic diseases, high BMI, low PSSS score, low GSES score are risk factors for psychological distress in obese patients during weight loss (*p* < 0.05). Clinical nurses, physicians, and health care personnel should pay attention to young female patients with obesity. For those patients who have chronic diseases such as hypertension, diabetes, and coronary heart disease, multi-team collaboration should be carried out to develop personalized weight loss plans for the patients and provide them with comprehensive support. Guide the patients to develop regular eating habits, ensure adequate sleep. Ensure sufficient physical activity every day. By learning professional weight loss knowledge, complete the weight loss goals regularly and maintain a good mindset. The nomogram model developed in this study indicates that when the incidence rate of psychological distress in obese patients is ≥0.396, there is a high possibility that the patients have psychological distress. We should pay attention to their psychological state and formulate corresponding intervention plans for the patients, thereby reducing the risk of psychological distress for the patients. Psychological distress is a constantly changing dynamic process, healthcare providers should establish health records for obese patients and conduct at least one follow-up visit every 3 months to timely understand the patients’ psychological conditions, identify high-risk groups in advance, and through effective intervention measures, improve the weight loss effect and quality of life of the patients.

### Limitations

Firstly, this study only included obese patients from tertiary hospitals in Jiangxi Province for sampling investigation, and it cannot fully represent the psychological distress levels of obese patients in other provinces of China. Therefore, in the later stage, further sampling surveys need to be conducted in other provinces of China to verify the predictive performance of the model. Secondly, only one method was used to build the model. It was suggested that in the future, various methods such as artificial neural networks and decision trees can be employed to construct the optimal prediction model.

## Data Availability

The original contributions presented in the study are included in the article/supplementary material, further inquiries can be directed to the corresponding authors.

## References

[1] Engin A. The definition and prevalence of obesity and metabolic syndrome: correlative clinical evaluation based on phenotypes. Adv Exp Med Biol. (2024) 1460:1–25. doi: 10.1007/978-3-031-63657-8_1 39287847

[2] Kundi H Amin ZM Friedman M Hagan K Al-Kindi S Javed Z . Association of obesity with psychological distress in young adults: patterns by sex and race or ethnicity. JACC Adv. (2024) 3:101115. doi: 10.1016/j.jacadv.2024.101115 39156117 PMC11327462

[3] NCD Risk Factor Collaboration (NCD-RisC). Worldwide trends in underweight and obesity from 1990 to 2022: a pooled analysis of 3663 population-representative studies with 222 million children, adolescents, and adults. Lancet. (2024) 403:1027–50. doi: 10.1016/S0140-6736(23)02750-2 38432237 PMC7615769

[4] World Obesity Federation. World Obesity Atlas 2025. London: World Obesity Federation (2025).

[5] Liu C Yuan YC Guo MN Xin Z Chen GJ Ding N . Rising incidence of obesity-related cancers among younger adults in China: a population-based analysis (2007-2021). Med. (2024) 5:1402–1412.e1402. doi: 10.1016/j.medj.2024.07.012 39181132 PMC11560649

[6] Jiang Z Wang Y Zhao X Cui H Han M Ren X . Obesity and chronic kidney disease. Am J Physiol Endocrinol Metab. (2023) 324:E24–e41. doi: 10.1152/ajpendo.00179.2022, PMID: 36383637

[7] Hachuła M Kosowski M Zielańska K Basiak M Okopień B. The impact of various methods of obesity treatment on the quality of life and mental health-a narrative review. Int J Environ Res Public Health. (2023) 20:2122. doi: 10.3390/ijerph20032122, PMID: 36767489 PMC9915720

[8] Steptoe A Frank P. Obesity and psychological distress. Philos Trans R Soc Lond Ser B Biol Sci. (2023) 378:20220225. doi: 10.1098/rstb.2022.0225 37661745 PMC10475872

[9] Fields ND Whitcomb BW Bertone-Johnson ER Martínez AD VanKim NA. Race-specific associations between psychological distress and obesity: the role of social cohesion. Ethn Health. (2023) 28:446–57. doi: 10.1080/13557858.2022.2052713, PMID: 35289677 PMC9475492

[10] Tan EJ Raut T Le LK Hay P Ananthapavan J Lee YY . The association between eating disorders and mental health: an umbrella review. J Eat Disord. (2023) 11:51. doi: 10.1186/s40337-022-00725-4, PMID: 36973817 PMC10044389

[11] Fulton S Décarie-Spain L Fioramonti X Guiard B Nakajima S. The menace of obesity to depression and anxiety prevalence. Trends Endocrinol Metab. (2022) 33:18–35. doi: 10.1016/j.tem.2021.10.005, PMID: 34750064

[12] Kornelius E Huang JY Lo SC Huang CN Yang YS. The risk of depression, anxiety, and suicidal behavior in patients with obesity on glucagon like peptide-1 receptor agonist therapy. Sci Rep. (2024) 14:24433. doi: 10.1038/s41598-024-75965-2, PMID: 39424950 PMC11489776

[13] Ngenge S Xie L McAdams C Almandoz JP Mathew MS Schellinger JN . Depression and anxiety as predictors of metabolic and bariatric surgery completion among ethnically diverse patients. Obes Surg. (2023) 33:2166–75. doi: 10.1007/s11695-023-06652-w, PMID: 37217806 PMC10202355

[14] Almhmoud H Alatassi L Baddoura M Sandouk J Alkayali MZ Najjar H . Polycystic ovary syndrome and its multidimensional impacts on women's mental health: a narrative review. Medicine. (2024) 103:e38647. doi: 10.1097/MD.0000000000038647, PMID: 38905372 PMC11191963

[15] Wong MCS Huang J Wang J Chan PSF Lok V Chen X . Global, regional and time-trend prevalence of central obesity: a systematic review and meta-analysis of 13.2 million subjects. Eur J Epidemiol. (2020) 35:673–83. doi: 10.1007/s10654-020-00650-3, PMID: 32448986 PMC7387368

[16] Sharp P Oliffe JL Kealy D Rice SM Seidler ZE Ogrodniczuk JS. Social support buffers young men's resilient coping to psychological distress. Early Interv Psychiatry. (2023) 17:784–91. doi: 10.1111/eip.13371, PMID: 36639361 PMC10946545

[17] Zajac IT Rice S Proeve M Kealy D Oliffe JL Ogrodniczuk JS. Suicide risk, psychological distress and treatment preferences in men presenting with prototypical, externalising and mixed depressive symptomology. J Ment Health. (2022) 31:309–16. doi: 10.1080/09638237.2020.1755026, PMID: 32401094

[18] Plasonja N Brytek-Matera A Décamps G. Psychological profiles of treatment-seeking adults with overweight and obesity: a cluster analysis approach. J Clin Med. (2022) 11:1952. 35407559 10.3390/jcm11071952 PMC8999798

[19] Shourabi E Vagharseyyedin SA. Relation among hope, self-efficacy, and psychological distress in hemodialysis patients: a path analysis. BMC Psychol. (2025) 13:528. doi: 10.1186/s40359-025-02848-0, PMID: 40394676 PMC12093832

[20] Zhu LH Shen YF Ren Q Lin J. Construction of a risk prediction model for the occurrence of acute skin failure in critically ill patients: a prospective study. J Nurs Res. (2024) 32:e338. doi: 10.1097/jnr.0000000000000627, PMID: 39046359

[21] Liu Y Zhang L Guo N Jiang H. Postpartum depression and postpartum post-traumatic stress disorder: prevalence and associated factors. BMC Psychiatry. (2021) 21:487. doi: 10.1186/s12888-021-03432-7, PMID: 34610797 PMC8491367

[22] Lukoševičiūtė J Šmigelskas K. Causal item of brief illness perception questionnaire (BIPQ) scale: the main categories. Health Psychol Res. (2020) 8:8485. doi: 10.4081/hpr.2020.8485, PMID: 32529089 PMC7270637

[23] Brown A Sergent H Vu AH Liu H Fisher J Somoza E . Sleeve-to-bypass conversion vs. sleeve-with-adjuvant GLP-1 receptor agonists: an academic multicenter retrospective study. Surg Endosc, (2025) 39:6155–62. 40691334 10.1007/s00464-025-11942-8

[24] Jamka M Popek J Bukowska-Posadzy A Mądry E Lisowska A Jończyk-Potoczna K . Psychological determinants of the effectiveness of conjugated linoleic acid supplementation in overweight and obese women-a randomized controlled trial. Front Nutr. (2024) 11:1342452. doi: 10.3389/fnut.2024.1342452, PMID: 39101007 PMC11294210

[25] Pigsborg K Kalea AZ De Dominicis S Magkos F. Behavioral and psychological factors affecting weight loss success. Curr Obes Rep. (2023) 12:223–30. doi: 10.1007/s13679-023-00511-6, PMID: 37335395

[26] Macho S Andrés A Saldaña C. Weight discrimination, BMI, or weight bias internalization? Testing the best predictor of psychological distress and body dissatisfaction. Obesity (Silver Spring). (2023) 31:2178–88. doi: 10.1002/oby.23802, PMID: 37424155

[27] Dakanalis A Mentzelou M Papadopoulou SK Papandreou D Spanoudaki M Vasios GK . The association of emotional eating with overweight/obesity, depression, anxiety/stress, and dietary patterns: a review of the current clinical evidence. Nutrients. (2023) 15:1173. doi: 10.3390/nu15051173, PMID: 36904172 PMC10005347

[28] Bray GA Ryan DH. Evidence-based weight loss interventions: individualized treatment options to maximize patient outcomes. Diabetes Obes Metab. (2021) 23:50–62. doi: 10.1111/dom.14200, PMID: 32969147

[29] Lambert DC Kane J Newberry C. Lifestyle therapy for obesity. Gastrointest Endosc Clin N Am. (2024) 34:577–89. doi: 10.1016/j.giec.2024.03.003, PMID: 39277292

[30] Gallo G Desideri G Savoia C. Update on obesity and cardiovascular risk: from pathophysiology to clinical management. Nutrients. (2024) 16:2781. doi: 10.3390/nu16162781, PMID: 39203917 PMC11356794

[31] Bianchettin RG Lavie CJ Lopez-Jimenez F. Challenges in cardiovascular evaluation and management of obese patients: JACC state-of-the-art review. J Am Coll Cardiol. (2023) 81:490–504. doi: 10.1016/j.jacc.2022.11.031, PMID: 36725178

[32] Powell-Wiley TM Poirier P Burke LE Després J-P Gordon-Larsen P Lavie CJ . Obesity and cardiovascular disease: a scientific statement from the American Heart Association. Circulation. (2021) 143:e984–e1010. doi: 10.1161/CIR.0000000000000973, PMID: 33882682 PMC8493650

[33] Jensen MT Nielsen SS Jessen-Winge C Madsen CMT Thilsing T Larrabee Sønderlund A . The effectiveness of social-support-based weight-loss interventions-a systematic review and meta-analysis. Int J Obes. (2024) 48:599–611. doi: 10.1038/s41366-024-01468-9, PMID: 38332127

[34] Jawara D Alagoz E Lauer KV Voils CI Funk LM. Exploring social support dynamics after bariatric surgery: insights from patients and providers. J Surg Res. (2024) 299:1–8. doi: 10.1016/j.jss.2024.03.047, PMID: 38677002 PMC11189728

[35] Bandura A. Social cognitive theory: an agentic perspective. Annu Rev Psychol. (2001) 52:1–26. doi: 10.1146/annurev.psych.52.1.1 11148297

[36] Palmeira AL Sánchez-Oliva D Encantado J Marques MM Santos I Duarte C . Motivational and self-efficacy reciprocal effects during a 12-month' weight regain prevention program. Br J Health Psychol. (2023) 28:467–81. doi: 10.1111/bjhp.12635, PMID: 36404726

